# Recent progress in the development of anti-malarial quinolones

**DOI:** 10.1186/1475-2875-13-339

**Published:** 2014-08-30

**Authors:** Richard M Beteck, Frans J Smit, Richard K Haynes, David D N’Da

**Affiliations:** Pharmaceutical Chemistry, School of Pharmacy, North-West University, Potchefstroom, 2520 South Africa; Centre of Excellence for Pharmaceutical Sciences, North-West University, Potchefstroom, 2520 South Africa

**Keywords:** Endochin, Quinolone, Decoquinate

## Abstract

Available anti-malarial tools have over the ten-year period prior to 2012 dramatically reduced the number of fatalities due to malaria from one million to less than six-hundred and thirty thousand. Although fewer people now die from malaria, emerging resistance to the first-line anti-malarial drugs, namely artemisinins in combination with quinolines and arylmethanols, necessitates the urgent development of new anti-malarial drugs to curb the disease. The quinolones are a promising class of compounds, with some demonstrating potent *in vitro* activity against the malaria parasite. This review summarizes the progress made in the development of potential anti-malarial quinolones since 2008. The efficacy of these compounds against both asexual blood stages and other stages of the malaria parasite, the nature of putative targets, and a comparison of these properties with anti-malarial drugs currently in clinical use, are discussed.

## Background

Malaria represents a significant global health threat, with 40% of the world’s population being at risk of contracting this disease. During 2012, nearly six-hundred and thirty thousand people died from the disease [1], with pregnant women and children under the age of five being the most vulnerable to infection [2]. By far the most (around 90%) deaths occur in sub-tropical and tropical Africa south of the Sahara (representing 564,300 of the total 627,000 deaths reported in 2012) [1], indicative of the endemic proportions that malaria has reached in this region.

**Figure 1:**
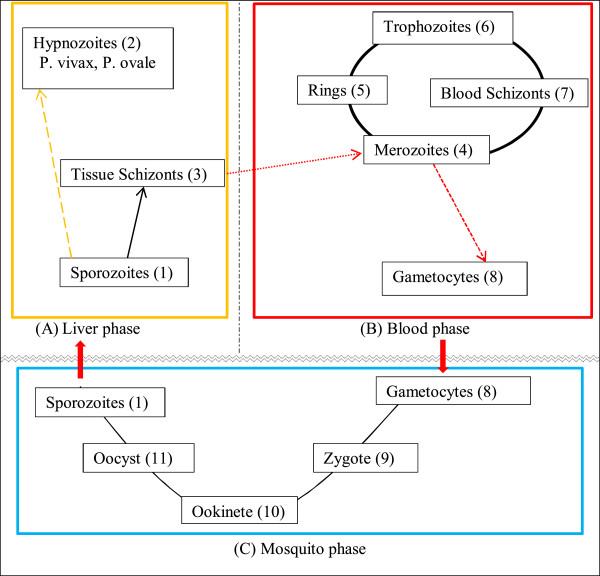
**Schematic life cycle of malaria parasite. (A)** Liver phase, **(B)** Blood phase, **(C)** Mosquito phase. The cycle progresses from **(A)** to **(B)**, and then to **(C)**.

Malaria arises from the invasion of red blood cells (RBCs) by a protozoan of the genus, *Plasmodium* [3]. Five species of the *Plasmodium* genus*,* i.e. *Plasmodium falciparum, Plasmodium ovale, Plasmodium vivax, Plasmodium malariae,* and *Plasmodium knowlesi* cause human malaria. Of these species, *P. falciparum* is responsible for the most severe form of malaria [4]. The malaria parasite is transmitted to humans following the bite of an infected female *Anopheles* mosquito. The parasite has a complex life cycle, involving the vector and a vertebrate host. Figure 1 illustrates the malaria parasite life cycle that progresses through three different phases, with each phase comprising its own different stages.

The liver phase (A): following the bite of an infected *Anopheles* mosquito, sporozoites (1) (infectious stage) are introduced into the bloodstream of the victim (host), from where they migrate to the liver. In the liver, each sporozoite develops into a tissue schizont (3). In *P. ovale* and *P. vivax*, the sporozoites develop into hypnozoites (2), the dormant form responsible for the relapse of the disease, months after the initial infection.

The blood phase (B): when the tissue schizont (3) ruptures in the liver, merozoites are released into the bloodstream, where they invade the RBCs. Within the RBCs, each merozoite transforms into a trophozoite (6) and later into a blood schizont (7), which multiplies asexually, giving rise to 16–32 merozoites (4). When the infected RBCs rupture, merozoites are released into the bloodstream to further invade more RBCs and hence continue asexual multiplication. The clinical manifestations of the disease (e.g. fever and chills) appear during this phase. Some of these merozoites develop into gametocytes (8).

The mosquito phase (C): when a mosquito feeds on an infected person, it ingests gametocytes with the blood. The gametocytes undergo asexual reproduction within the mosquito’s mid-gut, producing thousands of sporozoites (1), which then migrate into the salivary glands of the mosquito, from where they are injected into humans during a blood meal.

Hitherto, chemotherapy has remained the sole option for malaria treatment [5]. Quinine, an alkaloid present in the bark of Cinchona trees, was the first effective treatment for malaria [6]. Once the structure of quinine had been established by Rabe *et al.* [7], the syntheses of quinine analogues became the next focus. This led to the discovery of the quinoline chloroquine (CQ) (Figure 1) and related compounds, such as amodiaquine and piperaquine. Other quinolines bearing a benzylic hydroxyl group as in the case of quinine were also prepared, the most important of which was mefloquine [8]. CQ turned out to be the most successful drug: it was cheap, relatively safe and was used for decades, before the parasite developed resistance to the drug. Structurally quite different drugs, as represented by Fansidar (a combination of sulphadoxine and pyrimethamine), have since been introduced, but unfortunately efficacy has also been impeded by the development of resistance [9].

**Figure 2:**
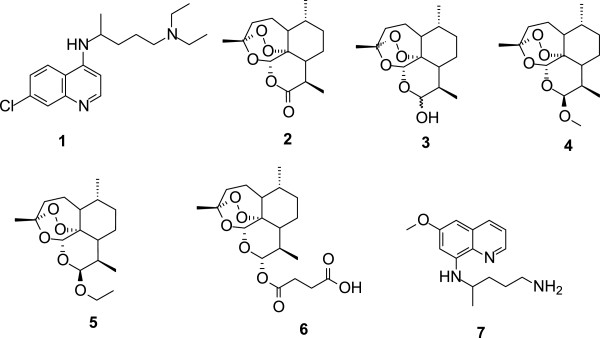
**Structures of chloroquine (1), artemisinin (2) and its derivatives: dihydroartemisinin (3), artemether (4), arteether (5), artesunate (6), and primaquine (7).**

Artemisinin and its derivatives (**2**, **3**–**6**, Figure 2), referred to as the ‘artemisinins’, are another class of anti-malarial drugs that are fast acting and potent against all resistant strains of the malaria parasite [10]. In an attempt to protect the artemisinins against the development of parasite resistance, the World Health Organization (WHO) recommended the use of these drugs in combination with other drugs, rather than in monotherapy. This led to the adoption of artemisinin based combination therapy (ACT) for the treatment of uncomplicated malaria in endemic countries. ACT combines an artemisinin derivative with a longer half-life anti-malarial drug. The rationale is that the fast acting artemisinin clears a larger proportion of the parasites within its short pharmacological half-life, whilst the longer half-life partner drug then continues the clearance as the artemisinin concentration falls to sub-therapeutic levels. In spite of this, tolerance to ACTs by the parasite has been reported in South-East Asia [11, 12], which is indicative of emerging resistance to the artemisinins. With the recent identification of genetic markers of the resistant phenotype and the pinpointing of the rapid spread of this phenotype, the search for new anti-malarial drugs becomes of utmost importance [13].

The anti-malarial drugs discussed above are more effective against the blood stage than any other stage of the malarial parasite. Primaquine is the only clinically proven drug that effectively kills hypnozoites (2) (the liver stage of the *P. vivax* parasite), and is also active against gametocytes (8) (the transmission stage of the parasite). Primaquine, however, causes fatal haemolysis in patients with glucose-6-phosphate dehydrogenase deficiency, an adverse side effect that has significantly limited its use [14].

Overall, resistance and tolerance associated with currently available anti-malarial drugs have created a driving force to the search for new chemical entities having novel modes of action, being readily available and meeting the Medicines for Malaria Venture (MMV) requirements for the next generation drugs needed to eradicate malaria. According to MMV, a suitable drug candidate for malaria eradication should be able to kill gametocytes, hypnozoites and other liver stages, thereby inhibiting transmission, relapse, as well as providing prophylaxis against the disease. Ideally, such a potential candidate should also have a minimum half-life of three days [15], although in practice such a property may be difficult to achieve.

## Review

One way of uncovering new compounds involves evaluating highly efficacious compounds in other therapeutic fields. It is apparent that some of these compounds contain the quinolone scaffold and are active against the malaria parasite.

## The quinolone scaffold

**Figure 3:**
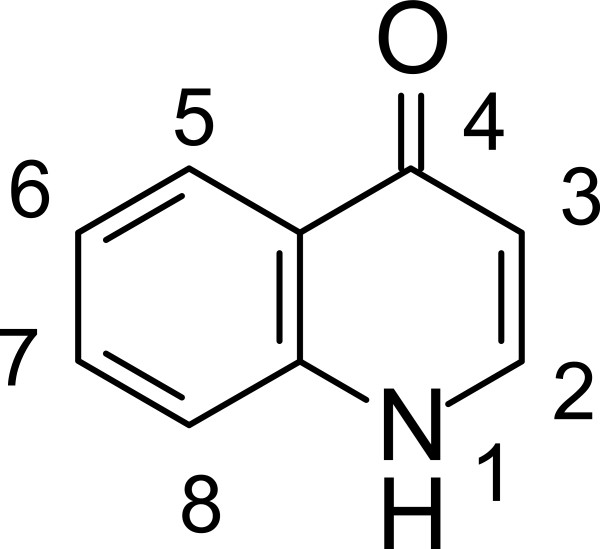
**The quinolone scaffold.**

Quinolones are synthetic compounds containing the 4-oxo-1,4-dihydroquinoline skeleton that may be written as the tautomeric 4-hydroxyquinoline (Figure 3). They are mostly used as antibiotics [16]. The first anti-bacterial quinolone, nalidixic acid, was discovered as a by-product during the synthesis of CQ [17]. Since the discovery of nalidixic acid and its anti-bacterial properties against gram negative bacteria, several structural modifications have been made to widen its spectrum of activity to also include gram positive bacteria [18].

Besides possessing bactericidal properties, the quinolone scaffold is present in the structures of certain anti-cancer [19] and anti-viral drugs [20], and also in anti-oxidants [21]. The scaffold is also incorporated into compounds that display anti-malarial activity [22].

Reports on the anti-malarial properties of quinolones, compared to their anti-bacterial properties, are relatively limited. Recent research involving the evaluation of anti-malarial properties of quinolones, however, indicates that these compounds demonstrate promising potential. They show very good efficacies and target more than one stage of the malaria parasite life cycle, including the blood, liver and gametocyte stages. They also seem to have novel modes of action, different from those of most of the current, clinically used drugs. Thus, in this review are presented details of anti-malarial quinolones documented within the past six years.

## Quinolones

### Endochin and its analogues

**Figure 4:**
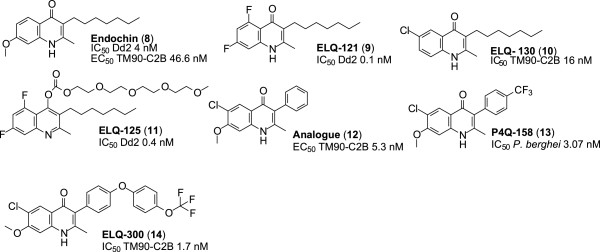
**Structures of endochin and its derivatives.**

The anti-malarial properties of endochin (**8**, Figure 4) have been known since 1948, when its activity against avian malaria was demonstrated [23]. Further research on this molecule has established that it is active against both the liver (phase A, Figure 1) and blood (phase B, Figure 1) stages of the parasites. It targets the cytochrome *bc*_*1*_ complex of the parasite [22]. However, endochin has proven to be ineffective *in vivo* against human malaria [24]. Its failure in humans has been ascribed to the fact that endochin is easily metabolized to inactive metabolites in the presence of cytochrome P450 (CYP450) enzymes [25].

Recent advances in understanding of both the drug mechanism of action and the manner in which resistance may arise suggest that previously abandoned lead molecules may in fact be viable anti-malarial drug candidates. This awareness has led to both a re-examination of many abandoned molecules, including quinolone endochin, and the activation of synthetic campaigns, aimed at generating more robust and potent analogues. These activities are exemplified by the development of the following new endochin analogues that have improved therapeutic properties, compared to the parent endochin.

#### 2-Methyl-3-(n-heptyl)-5,7-difluoroquinolone (ELQ-121)

Replacement of the methoxyl group at C-7 by fluorine and the insertion of fluorine at C-5 result in the compound, ELQ-121 (**9**, Figure 4). It has a half-maximal inhibitory concentration (IC_50_) value of 0.1 nM against the CQ sensitive (D6) and CQ resistant (Dd2) strains of *P. falciparum*. It is stable in the presence of microsomal elements (CYP450). The parent endochin has an IC_50_ value of 4 nM against the D6 and Dd2 strains and is unstable in the presence of CYP450 enzymes [25]. Thus, relative to endochin, the derivative ELQ-121 shows an approximate forty-fold improvement in IC_50_ for the inhibition of *P. falciparum in vitro* and exhibits an enhanced metabolic stability.

#### 2-Methyl-3-(n-heptyl)-6-cloroquinolone (ELQ-130)

Insertion of chlorine at C-6 results in the compound, ELQ-130 (**10**, Figure 4). It has an IC_50_ value of 16 nM against the TM90-C2B strain of *P. falciparum*. Although this is a less potent IC_50_ value compared to endochin (IC_50_ value of 11 nM against the same strain), ELQ-130 has enhanced metabolic stability than endochin and is devoid of cross-resistance with atovaquone [25].

#### 4-(2,5,8,11-Tetraoxatridecan-13-yl-carbonate)-2-methyl-3-(n-heptyl)-5,7-difluorquinolone (ELQ-125)

ELQ-125 (**11**, Figure 4) is a polyethylene glycol (PEG) conjugate of ELQ-121. It has, in addition to the two fluorine atoms at positions 5 and 7, a PEG moiety linked to the oxygen of the quinolone nucleus. It has an IC_50_ value of 0.4 nM against the D6 and Dd2 strains and is hence ten-fold (0.4 nM *vs* 4 nM) more active than the parent endochin. ELQ-125 displays higher oral bio-availability than both ELQ-121 and endochin. This property is attributed to the presence of the PEG moiety, which also enhances the aqueous solubility of the compound. As such, ELQ-125 does not have the solubility problem of endochin and its other analogues. ELQ-125 completely removed parasites from the blood stream of mice infected with *Plasmodium yoelii* on the third day of treatment, at a dose of 50 mg/kg/day, whereas endochin showed no activity in infected mice [25].

#### 3-Phenyl-4(1H)-quinolone

This analogue (**12**, Figure 4) has the *n*-heptyl chain at C-3 of endochin replaced by a phenyl ring, whilst a chlorine atom replaces the H atom at C-6. These modifications confer better aqueous solubility and microsomal stability relative to the parent endochin. This compound has an EC_50_ value of 15.3 nM against the TM90-C2B strain of *P. falciparum in vitro*. Compound **12** is therefore more potent than endochin, with an EC_50_ value of 46.6 nM against the same strain. It is important to note that this analogue shows no cross-resistance with atovaquone, contrary to endochin [26]. More importantly*,* results from *in vivo* studies indicated that the compound completely prevented exflagellation of microgametocytes at a dose of 10 μM, administered for fourteen days post infection. As such, this analogue has the same transmission blocking potential as primaquine [27].

#### 6-Chloro-7-methoxy-2-methyl-3-[4-(trifluoromethyl)phenyl]quinolin-4(1H)-one (P4Q-158)

In P4Q-158 (**13**, Figure 4), the *n*-heptyl chain of endochin is replaced with a trifluorotoluene moiety at C-3, whereas a chlorine atom replaces the H atom at C-6. P4Q-158 has potent anti-malarial activity *in vitro*, with an IC_50_ value of 3.07 nM against the liver stage of the *Plasmodium berghei* parasite. However, its potency is slightly less than that of atovaquone that has an IC_50_ value of 1.42 nM against the liver stage of *P. berghei.* P4Q-158 also demonstrates strong activity against the liver stage of *P. berghei in vivo*, as mice treated with this compound at a dose of 10 mg/kg displayed a more than 60% survival rate, compared to untreated mice [28].

#### 6-Chloro-7-methoxy-2-methyl-3-{4-[4-(trifluoromethyl)phenoxy]phenyl}quinolin-4(1H)-one (ELQ-300)

ELQ-300 (**14**, Figure 4) has a diaryl ether moiety replacing the C-3 *n*-heptyl substituent of endochin. Besides being metabolically more stable than endochin, ELQ-300 exhibits potent *ex vivo* anti-malarial activity against MDR *P. falciparum*, having IC_50_ values of 1.8 nM and 1.7 nM against the W2 and TM90-C2B strains, respectively. The drug is thus substantially more potent than CQ that has IC_50_ values of 126 nM and 96.2 nM against the W2 and TM90-C2B strains respectively.

Furthermore, ELQ-300 shows no cross-resistance with atovaquone, displays a high selectivity for the parasite respiratory *bc*_*1*_ complex (selectivity index ≥ 20,000), and is very potent against the early and late stages of *P. falciparum* gametocytes. At a concentration of 0.1 μM, ELQ-300 completely stopped further development of stage I and II gametocytes, and it is active against stage IV gametocytes, with an IC_50_ value of 79.1 nM. It also has an ED_50_ value of 0.02 mg/kg/day against murine *P. yoelii*. At a dose of 0.3 mg/kg/day, administered for 30 days post infection, ELQ-300 completely cleared parasites in infected murine models [29]. Presently, ELQ-300 is undergoing formulation studies in preparation for clinical studies [30].

#### Hydroxy-2-dodecyl-4-(1H)-quinolone (HDQ) and its analogues

**Figure 5:**
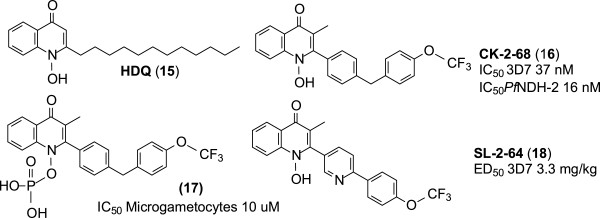
**Structure of HDQ and its derivatives.**

HDQ (**15**, Figure 5) is the only compound known to inhibit both the NADH:ubiquinone oxidoreductase enzyme (*Pf*NHD-2) and the *bc*_*1*_ complex in the respiratory chain of *P. falciparum*. This multi-target inhibition confers a benefit over the single target inhibition in that the former delays the onset of drug resistance [31]. Because of this advantage, HDQ had been used as a starting point for a drug discovery project that has led to several new quinolone analogues with promising anti-malarial activities. Examples of the most effective of these analogues are discussed next.

#### 7-Chloro-3-methyl-2-{4-[4-(trifluoromethoxy)benzyl]phenyl}quinolin-4(1H)-one (CK-2-68)

This HDQ derivative (**16**, Figure 5) bears a [4-(4-trifluoromethoxy)benzyl]phenyl group at C-2 of the quinolone nucleus instead of the metabolically vulnerable *n*-dodecyl unit present in HDQ. This 2-bisaryl quinolone has an IC_50_ of 31 nM against the 3D7 strain of *P. falciparum in vitro*. When given orally at a concentration of 20 mg/kg, CK-2-68 completely cleared *P. berghei* parasites in mice. It is stable in the presence of human microsomal elements. It inhibits NADH:ubiquinone oxidoreductase of *P. falciparum* with an IC_50_ value of 16 nM. This compares to a value higher than 1,000 nM for atovaquone. CK-2-68 and its phosphate pro-drug (**17**, Figure 5) have activities against *P. falciparum* microgametocytes similar to that of atovaquone (IC_50_ ~ 10 μM) [31–33] *.*

#### 7-Fluoro-3-methyl-2-{6-[4-(trifluoromethoxy)phenyl]pyridin-3-yl}quinolin-4(1H)-one (SL-2-64)

In SL-2-64 (**18**, Figure 5)*,* a 2-[(4-trifluoromethoxy)benzyl)]pyridine moiety replaces the *n*-dodecyl unit at C-2 in HDQ. The pyridine unit in this molecule lowers lipophilicity and increases aqueous solubility relative to CK-2-68. SL-2-64 has activity comparable to that of some clinically used anti-malarial drugs against the 3D7 strain of *P. falciparum. In vivo*, this derivative has an ED_50_ value of 3.3 mg/kg against the 3D7 strain in murine models. This activity is similar to that of artemether, which has an ED_50_ value of 3.1 mg/kg against the same strain. This HDQ analogue is listed in the MMV discovery pipeline [31].

### Acridinones

**Figure 6:**
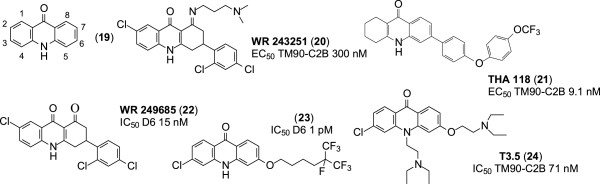
**Structure of acridinone and analogues.**

Acridinones are tricyclic compounds incorporating the 4-oxo-1,4-dihydroquinolone skeleton, and are thus structurally closely related to quinolones. The anti-malarial activity of a compound containing the acridinone scaffold (**19**, Figure 6) was reported in 1947 [34]. This compound garnered little interest until 1970 when the anti-malarial prophylactic property of floxacrine came (an analogue of acridinone) was discovered [35]. Shortly after floxacrine came, WR 243251 (**20**, Figure 6) with improved therapeutic properties. However, these compounds were not further evaluated, due to their poor aqueous solubility and metabolic instability [36]. Recent studies aimed at re-evaluating the acridinone scaffold through structure–property relationships (SPR) and optimization by using structure-activity relationships (SAR) have led to the discovery of promising anti-malarial analogues.

#### 6-{4-[4-(Trifluoromethyl)phenoxy]phenyl}-1,3,4,4a,9a,10-hexahydroacridin-9(2H)-one (THA 118)

THA 118 (**21**, Figure 6) has a diphenyl ether moiety replacing the phenyl moiety at C-6 in WR 243251. THA 118 possesses more potent anti-malarial activity and has improved metabolic stability and solubility relative to WR 243251. Indeed, THA 118 displays anti-malarial activity *in vitro* with EC_50_ values of 12.2 nM and 9.1 nM against the W2 and TM90-C2B strains of *P. falciparum* respectively, compared to WR 243251, which has EC_50_ values of 25.0 nM and 300.0 nM against W2 and TM90-C2B strains [37].

#### 7-Chloro-3-(2,4-dichlorophenyl)-3,4-dihydroacridine-1,9(2H,10H)-di-one (WR 249685)

This analogue (**22**, Figure 6) possesses potent *in vitro* anti-malarial activity (IC_50_ 15 nM) comparable to that of CQ (IC_50_ 7.4 nM) against the D6 strain of *P. falciparum* [38]. WR 249685 has a higher selectivity index for the parasite *bc*_*1*_ complex than does atovaquone. This was established by comparing their *in vitro* therapeutic indices (IVTI) for the human *bc*_*1*_ complex, i.e. IVTI was 4,600 for WR 249685 and 24 for atovaquone. In addition to targeting the parasite *bc*_*1*_ complex, WR 249685 also binds to haem, although it has a low affinity for haem compared to CQ [39]. Thus, it is unlikely to exert an anti-malarial effect in this manner relative to CQ.

### Haloalkoxyacridinones

This is a relatively new class of acridinone [38]. The most active analogues in this class have at C-3 of the acridone ring an alkoxy moiety terminating in one or more trifluoromethyl units. The acridinone (**23**, Figure 6) is an example of such an analogue. It exhibits extraordinarily strong anti-malarial activity *in vitro*, with IC_50_ values of 1 pM against both the D6 and Dd2 strains of *P. falciparum*. With such favourable IC_50_ values, this compound appears to be the most effective anti-malarial ever synthesized and tested in a laboratory. Although its mode of action is still unknown, it is assumed that it binds to the *bc*_*1*_ complex due to its structural similarity with known anti-malarial drugs targeting that complex. This acridinone analogue has an *in vitro* therapeutic index higher than 100,000 [39] and has been patented in the US as an anti-malarial drug [40].

#### 3-Chloro-6-(2-diethylamino-ethoxy)-10-(2-diethylamino-ethyl)-acridinone (T3.5)

This 10-*N* substituted acridinone analogue (**24**, Figure 6) incorporates two potential anti-malarial features, namely, a haem-targeting unit through the tricyclic acridone core, and a quinoline resistance-reversal unit, due to the *N*-(2-diethylamino-ethyl) unit. T3.5 has *in vitro* IC_50_ values of 77.3 nM and 71.3 nM against the Dd2 and TM90-C2B strains of *P. falciparum,* respectively. In comparison, CQ has IC_50_ values of 124.7 nM and 122.7 nM against the same strains respectively. This acridinone analogue is non-toxic to mammalian cell lines both *in vitro* and *in vivo* and exhibits synergism with each of piperaquine, CQ and amodiaquine against the Dd2 strain [41].

### Carboxyquinolones

**Figure 7:**
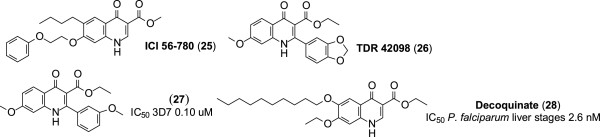
**Structures of carboxylquinolones.**

Since the discovery of the anti-malarial activity of ICI56-780 (**25**, Figure 7) [42], recent research on quinolone anti-malarial drugs has focused on carboxyl derivatives of quinolones. Due to the need for cheap anti-malarial drugs that have novel modes of action, commercially available compounds with antiparasitic activity had been screened for anti-malarial activity. This has led to the discovery of various 3-carboxylquinolones as potential anti-malarial drugs.

#### Ethyl 2-(1,3-benzodioxol-5-yl)-7-methoxy-4-oxo-1,4-dihydroquinoline-3-carboxylate (TDR 42098)

This compound demonstrates improved potency against the blood stages of the CQ-R K1 and CQ-S NF54 strains of *P. falciparum*, and has better physicochemical properties, than endochin analogues, bearing a lipophilic side chain [43]. Derivatization of TDR 42098 (**26**, Figure 6) produced the analogue (**27**, Figure 7) bearing a meta-substituted aromatic ring at C-2. This derivative has mid-range EC_50_ values of 0.13 μM and 0.10 μM against the K1 and 3D7 strains of *P. falciparum,* respectively [44].

#### 6-Deoxy-7-ethoxy-4-oxo-1H-quinoline-3-carboxylic acid ethyl ester (decoquinate (DQ))

The anti-malarial activities of this long used anti-coccidial drug were recently discovered during screening conducted against the liver stages of *P. falciparum. In vitro*, decoquinate (DQ) (**28**, Figure 7) is potent against the liver (IC_50_ 2.6 nM), blood (IC_50_ 10 nM), and sexual (IC_50_ 36 nM) stages of the malaria parasite. It reportedly targets the *bc*_*1*_ complex of *P. falciparum* and shows no cross-resistance with atovaquone [45, 46].

**Figure 8:**
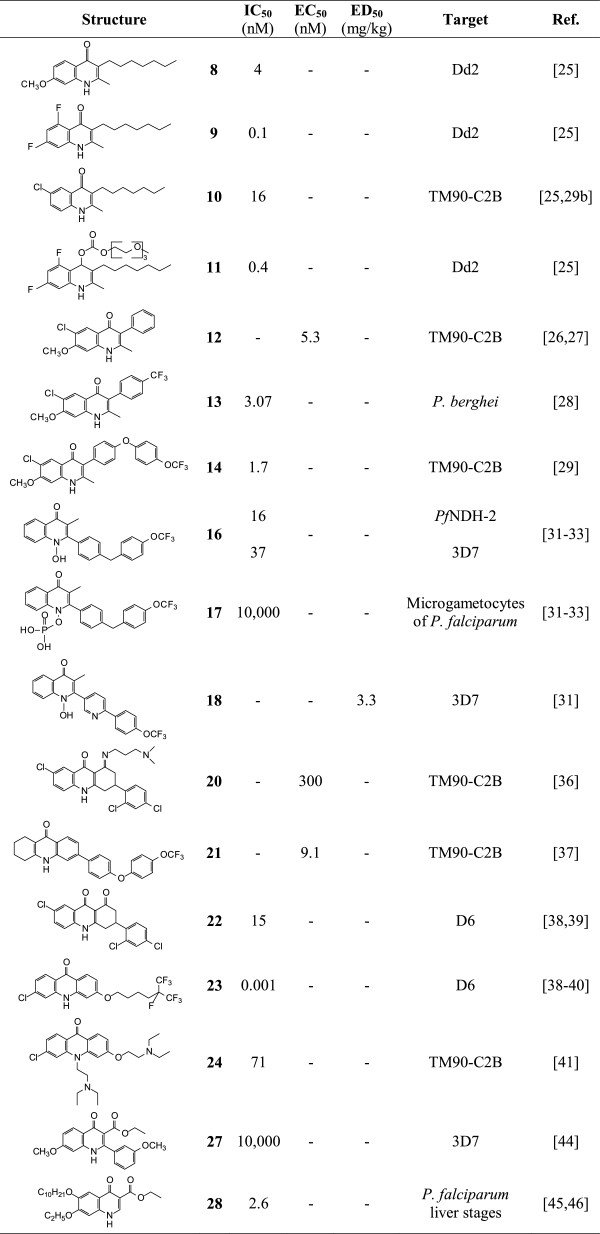
**All structures, and their potencies.**

According to the results of the above studies, it is apparent that DQ meets the MMV requirements for the next generation drugs needed for malaria eradication [15]. Despite demonstrating good anti-malarial activity, DQ has the major liability of poor aqueous solubility. It also undergoes facile metabolism to the free carboxylic acid. However, DQ is a very cheap compound and is readily available. Thus, it is apparent that new derivatives of DQ can be prepared through reducing the lipophilicity, enhancing both the solubility and metabolic stability by replacing the ester group by appropriate amide groups.The important features of each compound are summarized in Figure 8.

## Conclusion

The quinolone nucleus is a chemotype common to classes of chemotherapeutic agents including antibiotic, anti-viral and anti-cancer drugs. More recent research on the anti-malarial activities of quinolones indicates that such compounds are relatively potent against the blood, liver and transmission stages of the malarial parasite and act on one or more targets of the parasite. These findings overall indicate the importance of the quinolone nucleus in the development of drugs aimed at eradicating malaria. Nevertheless, more research is required in order to address the specific difficulties associated with quinolone lead compounds, including those of relatively poor aqueous solubility and metabolic instability.

## Abbreviations

WHO: World Health Organization
MMV: Medicines for Malaria Venture
RBC: Red blood cell
CQ: Chloroquine
ACT: Artemisinin combination therapy
IC_50_: Half maximal inhibitory concentration
ED_50_: Half maximal effective dose
EC_50_: Half maximal effective concentration
HDQ: Hydroxy-2-dodecyl-4-(1H)-quinolone
DQ: Decoquinate
SPR: Structure property relationship
SAR: Structure activity relationship
IVTI: *In vitro* therapeutic index
nM: Nano-molar
μM: Micro-molar
USPTO: United States Patent and Trademark Office.
